# New Serious Safety Warnings for Targeted Anticancer Agents After Their Initial FDA Approval

**DOI:** 10.3390/cancers17040584

**Published:** 2025-02-08

**Authors:** Dimitar Stefanovski, Damjan Manevski, Domen Ribnikar, Boštjan Šeruga

**Affiliations:** 1 Division of Medical Oncology, Institute of Oncology Ljubljana, Zaloška Cesta 2, 1000 Ljubljana, Slovenia; dstefanovski@onko-i.si (D.S.); dribnikar@onko-i.si (D.R.); 2 Faculty of Medicine, University of Ljubljana, Vrazov Trg 2, 1000 Ljubljana, Slovenia; 3 Institute for Biostatistics and Medical Informatics, Faculty of Medicine, University of Ljubljana, Vrazov Trg 2, 1000 Ljubljana, Slovenia; damjan.manevski@mf.uni-lj.si

**Keywords:** adverse drug reaction, targeted anticancer agent, updated drug label

## Abstract

**Background:** New safety concerns about targeted anticancer agents (TAAs) often emerge in the first few years after their initial regulatory approval. Our aim was to determine whether new serious and potentially fatal adverse drug reactions (ADRs) continue to emerge in the updated drug labels of TAAs several years after their initial regulatory approval and whether their emergence can be predicted. **Methods:** The updated drug labels of TAAs approved by the U.S. Food and Drug Administration before July 2013 were analyzed. Serious and potentially fatal ADRs were identified in the Warnings & Precautions (WPs) and Boxed Warnings (BWs) sections of the updated drug labels. Generalized linear mixed models were used to examine the associations between the number of adverse drug reactions and time, drug type (small molecules vs. monoclonal antibodies), and the availability of companion diagnostics for biomarkers. **Results:** Among 37 eligible TAAs, 25 (68%) were small molecules and 11 (30%) had available companion diagnostics for the biomarkers. Time was a significant predictor of new WPs (*p* ˂ 0.001) and BWs (*p* = 0.008). The updated drug labels of the small molecules received significantly more new WPs (*p* = 0.042) as compared to monoclonal antibodies. The availability of the companion diagnostics for the biomarkers did not have an impact on the emergence of new ADRs. **Conclusions:** New serious ADRs of TAAs continue to emerge in updated drug labels several years after their initial regulatory approval. Oncologists, regulators, and payers should be aware of the changing risk–benefit ratios of approved TAAs.

## Simple Summary

Knowledge about the tolerability of targeted anticancer agents can improve after their regulatory approval. In this study, we analyzed the emergence of new serious safety warnings in the updated drug labels of targeted anticancer agents that were marketed for at least 10 years. Additionally, possible predictors for their emergence were studied. We found that new serious safety warnings have continued to emerge several years after the initial approval of the targeted anticancer agents, especially small-molecule targeted agents. This research highlights a need for ongoing safety monitoring of targeted anticancer agents. Oncologists, regulators, and payers should be aware of the changing risk–benefit ratios of approved targeted anticancer agents.

## 1. Introduction

Treatment with targeted anticancer agents (TAAs) improves the survival and quality of life of patients with various cancer types. Unlike chemotherapy (ChT), TAAs specifically interfere with different molecular pathways involved in the growth and progression of malignant cells and are generally considered to be less toxic than ChT [1]. The precise targeting of some approved TAAs can be achieved through the identification of biomarkers, which can be detected using in vitro companion diagnostic tests for biomarkers (hereafter CDxs) [2]. However, patients treated with TAAs may experience a spectrum of toxicities, unique to this class of agents, with safety profile differences between small molecules (SMs) and monoclonal antibodies (mAbs) [3]. Moreover, previous research has shown that TAAs without specific molecular targets are more toxic than those with specific targets identified with a CDx [4].

Despite rigorous documentation of adverse events, some serious drug-related toxicities (i.e., adverse drug reactions [ADRs]) of TAAs may remain unrecognized during clinical trials but become evident only when those TAAs are used in everyday clinical practice [5]. The main reasons may be the relatively small number of patients who participate in pivotal clinical trials and the very restrictive eligibility criteria for their participation [6]. Furthermore, symptomatic ADRs that are experienced by patients cannot be assessed reliably by investigators and may be under-reported within clinical trials [7]. Throughout the post-market phase, regulatory agencies meticulously compile safety data on approved anticancer agents from diverse sources, including ongoing clinical trials, spontaneous reporting, phase IV trials, and surveillance programs [8,9]. Previous research showed that 49% and 55% of serious and potentially fatal ADRs of TAAs, respectively, reported in updated drug labels during the first few years after their regulatory approval, were not described in the initial drug labels at the time of their approval [10]. Furthermore, an assessment of post-market serious safety signals within two years of the U.S. Food and Drug Administration (FDA) approval of new cancer drugs showed that drugs with accelerated approval had a greater incidence of substantial safety-related changes as compared with those with regular approval [11]. The long-term trajectory of emerging new serious ADRs in the updated drug labels of TAAs and the possible predictors for their emergence are currently not known.

Here, we have hypothesized that new serious and potentially fatal ADRs continue to emerge in the updated drug labels of TAAs several years after their initial approval. We also investigated potential predictors for the emergence of new ADRs in the updated drug labels of TAAs, including drug type and the availability of a CDx.

## 2. Methods

### 2.1. Identification of Targeted Anticancer Agents and Analysis of Adverse Drug Reactions

In August 2023, we identified all TAAs approved for the treatment of solid cancers and hematological malignancies by the FDA before July 2013, ensuring that each eligible TAA had been on the market for at least 10 years. The initial and all the updated drug labels of the eligible TAAs were retrieved from the FDA Drug Approvals and Databases website [12]. For each particular anticancer agent, the sources of new data on safety concerns were not only clinical trials and real-world studies but also spontaneous reporting through various reporting systems. The drug labels of the approved TAAs were consulted for serious ADRs in the Warnings & Precautions (W&Ps) and for potentially fatal ADRs in the Boxed Warnings (BWs) sections of their drug labels, excluding embryo–fetal toxicity. ADRs reported in the W&Ps and BWs sections are considered serious and clinically significant, with clear causal links to the drugs. BWs highlight fatal, life-threatening, and permanently disabling adverse reactions, urging clinicians to carefully monitor and weigh the risks and benefits of the drugs [13].

### 2.2. Outcomes of Interest

The primary outcome of interest was a long-term trajectory of emerging new serious and potentially fatal ADRs in the updated drug labels of TAAs. A late ADR was defined as any new W&P or BW that first appeared in an updated drug label ≥5 years after the initial approval of the corresponding TAA. We also investigated potential predictors for the emergence of new ADRs in updated drug labels, such as type of TAA (SM vs. mAb) and availability of a CDx (yes vs. no). The availability of a CDx in this study was defined as the use of a CDx for all approved indications of a particular TAA.

### 2.3. Statistical Analysis

Descriptive statistics were used to summarize the data. The Mann–Whitney test was used for comparisons of the number of ADRs (i.e., W&Ps and BWs) concerning the type of TAA and availability of a CDx. To study a time trajectory of emerging new ADRs in updated drug labels concerning other variables, a generalized linear mixed model was used [14]. Since the outcome was a count (the number of ADRs over time), a negative binomial inverse link function, which accounted for overdispersion, was used. The effect of time was non-linear; thus, its quadratic component was added in the model. While drug type (SM vs. mAb) and the availability of a CDx (yes vs. no) were added as fixed effects, the TAA was added as a random effect into the model. The model also allowed for a random effect in the slope of time and time squared. Intercept independence and slope coefficients for time and time squared were incorporated. Inclusion of the interactions between the covariates was not necessary in the final model. Additionally, sensitivity analyses were conducted in which the availability of a CDx was defined as the use of a CDx for at least one indication of particular TAAs.

The analysis was performed using the R programming language, version 4.3.1. *p*-values of <0.05 were deemed statistically significant. No adjustments for multiple comparisons were made.

## 3. Results

### 3.1. Characteristics of Eligible Targeted Anticancer Agents

We identified 38 TAAs approved by the FDA between 1997 and 2013. One drug, tositumomab, was withdrawn from the market for commercial reasons in October 2013, and its labels were not available on the FDA website. Therefore, 37 TAAs were eligible for this analysis. The characteristics of the eligible TAAs are presented in Supplementary Table S1. Of these, 25 (68%) were SMs and 11 (30%) had an available CDx (i.e., drug labels recommended the use of a CDx for all approved indications). The drug labels of four (11%) TAAs recommended the use of a CDx for at least one but not all approved indications. Nineteen (51%) TAAs were approved before the year 2011. Overall, the median time from the regulatory approval to the last updated drug label was 11.8 years (IQR: [10.0–15.0]).

### 3.2. Reporting of Warnings & Precautions

**Figure 1:**
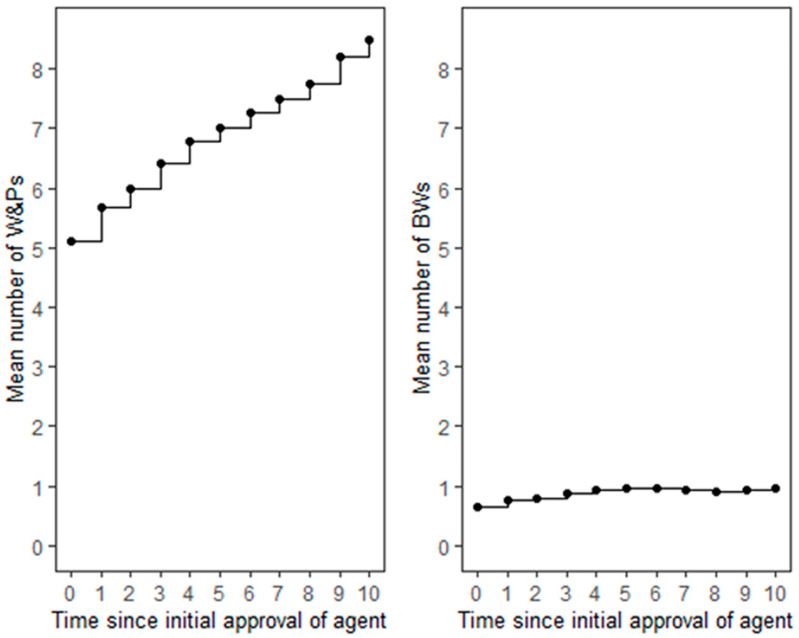
The mean numbers of Warnings & Precautions (W&Ps; **left**) and Boxed Warnings (BWs; **right**) as a function of time after initial approval.

**Figure 2:**
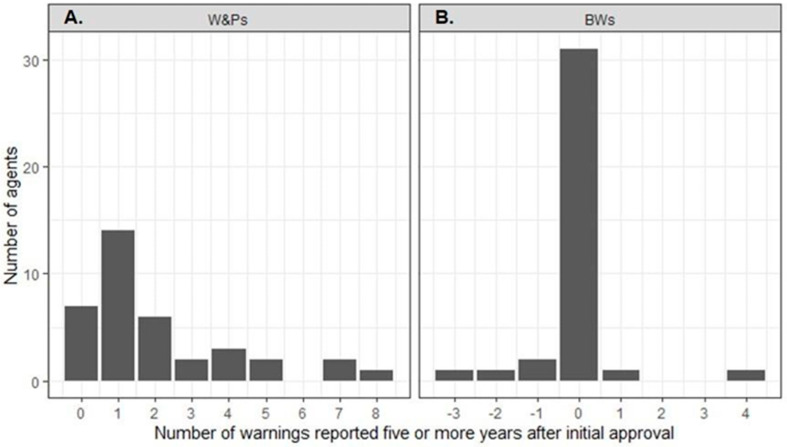
Distribution of Warnings & Precautions (W&Ps) (**A**) and Boxed Warnings (BWs) (**B**) for targeted anticancer agents, reported five or more years after their initial approval.

The mean number of W&Ps reported in the initial drug labels of the TAAs was 5.1. After initial approval, 95% (35/37) of the TAAs received at least one new W&P (Supplementary Figure S1). Over time, there was a consistent increase in the number of W&Ps for most agents, reaching a mean number of W&Ps of 8.5 at 10 years (Figure 1). Only two agents, ziv-aflibercept and pertuzumab, maintained a constant number of W&Ps throughout this period (Supplementary Figure S1). At least one late W&P was reported for 30 (81%) TAAs (Figure 2A). The highest numbers of new late W&Ps were reported for rituximab, imatinib mesylate, and sunitinib malate. In contrast, no new late W&Ps were reported for erlotinib hydrochloride, ofatumumab, pertuzumab, ziv-aflibercept, regorafenib, ponatinib hydrochloride, and ado-trastuzumab emtansine (Supplementary Figure S1). For nine (24%) TAAs, new W&Ps emerged more than 10 years after their initial regulatory approval (Supplementary Figure S1). There were significantly fewer new late W&Ps reported for the mAbs compared to the SMs (1.3 vs. 2.4; *p* = 0.03). Although there were fewer new late W&Ps for agents with an available CDx as compared with those without, the difference was not statistically significant (availability of a CDx, yes vs. no: 1.4 vs. 2.4; *p* = 0.19). Sensitivity analysis did not change these results.

The results of the generalized linear mixed model for the W&Ps are presented in Table 1. While time was a significant predictor for the increasing number of new W&Ps (coef. for time = 0.06; *p* ˂ 0.001), the rate of appearance of new W&Ps significantly decreased over time (coef. for time^2^ = −0.002; *p* = 0.001). The updated drug labels of the SMs received significantly more new W&Ps as compared to the mAbs over time (coef. for SMs = 0.35; *p*-value = 0.04). The number of W&Ps was smaller for TAAs with an available CDx as compared with those without an available CDx, although this difference was not statistically significant (coef. for CDx, yes = −0.15; *p*-value = 0.44). A sensitivity analysis did not change our results (Supplementary Table S2).

### 3.3. Reporting of Boxed Warnings

The overall number of BWs was substantially lower as compared to the W&Ps, with a mean of 0.65 BWs reported in the initial drug labels at the time of the regulatory approval of the TAAs. After initial approval, 37.8% (14/37) of the TAAs received at least one new BW (Supplementary Figure S2). Over time, the increase in the BWs was modest, reaching a mean of 0.97 ADRs at 10 years (Figure 1). Notably, 23 (62%) TAAs maintained a constant number of BWs throughout the entire period, and for 17 (46%) TAAs, the number of BWs remained at zero. While an increase in the number of BWs was observed for ten (27%) TAAs, a decrease in the number of BWs occurred for four (11%) agents—bevacizumab, cabozantinib s-malate, panitumumab, and ipilimumab (Supplementary Figure S2). At least one late new BW was reported in the drug labels of only two (5%) agents: rituximab and ibritumomab tiuxetan. For 31 (84%) TAAs, the number of BWs remained unchanged after five years (Figure 2B). The number of late BWs did not differ between drug types (*p* = 0.77) or depend on the availability of a CDx (*p* = 0.76).

The results of the generalized linear mixed model for the BWs are presented in Table 2. Similar to the W&Ps, time was a significant predictor of an increasing number of new BWs (coef. for time = 0.07; *p* = 0.008) but the rate of the appearance of new BWs decreased over time (coef. for time^2^ = −0.05; *p* = 0.01). The updated drug labels of the TAAs classified as SMs received a significantly smaller number of BWs as compared to the mAbs over time (coef. for SMs = −3.11; *p* < 0.001). Similar to the analysis of the W&Ps, there were no statistically significant differences in the number of BWs in regard to the availability of a CDx (coef. for CDx = 0.22; *p* = 0.77). These results did not differ in the sensitivity analysis (Supplementary Table S3).

## 4. Discussion

The process of the drug licensing and regulatory approval of new anticancer agents involves a careful balancing of benefits against risks. For various reasons, information on the safety of new anticancer agents is often limited at the time of their regulatory approval [6,10]. The results of our study showed that new serious and potentially fatal ADRs have continued to emerge in the updated drug labels of TAAs several years after their initial approval; for 81% and 5% of the TAAs, new W&Ps and BWs emerged five or more years after regulatory approval, respectively. However, the rate of appearance of new ADRs decreased over time. As compared to mAbs, the drug labels of SMs are more likely to receive new serious W&Ps.

After initial regulatory approval, TAAs often receive several additional, supplemental indications, leading to their use in a broader population of patients with different types of cancer. Supplemental indications accounted for 75% of new oncology approvals in 2018 [15]. In a cohort of cancer agents approved by both the FDA and the European Medicines Agency (EMA), manufacturers added supplemental indications at a higher rate in the U.S. as compared to the EU despite starting with nearly identical numbers of indications at the time of initial approval [16]. A study of the first and supplemental indications of drugs approved by the FDA and the EMA, among which the largest subset was anticancer agents, showed that the proportion of supplemental indications rated as having a high therapeutic value was substantially lower than that for first indications [17]. One of the possible factors contributing to the lower therapeutic value of the supplemental indications might be new ADRs that were not known at the time of initial drug approval but became evident later when these anticancer agents were being evaluated in a broader population of patients with cancer.

A safety assessment within single-arm trials (usually with smaller sample sizes and shorter follow-up than pivotal randomized clinical trials) may be unreliable, as the symptoms of advanced cancer can mimic and obscure ADRs [18]. Roughly 60% of the new TAA indications between 2012 and 2021 were based on the results of the early-phase clinical trials, with the annual approval rates based on them rising by 22.2% (vs. 5% from randomized clinical trials) [19]. This may result in potentially unreliable assessment of the benefit–risk profiles at the approval of new indications, and new ADRs may emerge only when these agents are used in randomized clinical trials and/or everyday clinical practice. Furthermore, the long-term and sequential use of various TAAs within and/or outside of clinical trials may also be associated with an increased risk of the development of new serious ADRs, especially in the metastatic setting, where TAAs are often administered for several months or years, sometimes even beyond disease progression [20,21]. Prolonged exposure to TAAs may reveal previously undetected ADRs, as the buildup of toxic substances in the body increases the likelihood of ADRs [22]. The design of registrational clinical trials and prolonged use of the TAAs may be important factors for the emergence of new serious ADRs after their regulatory approval.

Our findings showed that SMs have a higher likelihood of receiving new serious W&Ps as compared to mAbs. While small molecules are mostly intracellular kinase inhibitors, the majority of antibodies are extracellular receptor ligands, and differences in safety could arise from the very different mechanisms of action rather than the intrinsic properties of these two groups of agents. Both mAbs and SMs can exhibit on-target and off-target effects due to their interaction with common signaling pathways in cancer and normal cells. It is well-known that the toxic potential of SMs correlates positively with the number of inhibited kinases and that the off-target effects may increase the likelihood of ADRs [23,24,25,26]. In contrast to the SMs, mAbs exhibit greater selectivity and affinity for their targets, contributing to a lower incidence of new ADRs [27]. Moreover, while mAbs are administered parenterally, the oral administration of SMs leads to greater inter-individual variability of plasma concentrations and possible overexposure to the SMs at a given dose [28]. The plasma concentrations of the SMs can also be influenced by environmental and genetic factors due to their reliance on cytochrome P450 (CYP) enzyme metabolism and other pharmacokinetic processes [29,30]. Consequently, interactions with other drugs that are commonly used in patients in everyday clinical practice can significantly alter exposure to SMs [31]. In contrast, mAbs avoid CYP metabolism, resulting in fewer interactions and potentially lower toxicity. In our study, the availability of a CDx did not predict the emergence of serious and potentially fatal ADRs. The results of a meta-analysis evaluating the impact of a CDx on the efficacy and safety of TAAs corroborate our findings, as the availability of the CDx did not significantly affect the rate of toxic deaths; however, it reduced the odds for treatment discontinuation and grade 3-4 adverse events in this meta-analysis [32]. In summary, the off-target effects of TAAs, the variability in their metabolism due to genetic factors, and drug–drug interactions may all be important factors responsible for the development of new serious ADRs when these agents are used in everyday clinical practice. Future research should be focused on pharmacogenetics and dose-finding studies, which might lead to more predictable safety of newly approved TAAs, especially of SMs.

Our study has several limitations. First, the small number of TAAs might have impacted the robustness of our statistical findings, especially the findings about the impact of a CDx on the emergence of new ADRs. However, for all 37 agents, which have been marketed for at least 10 years, annual data were available; thus, we considered 588 observations when fitting each regression model. Alternatively, a meta-analytical approach would require an integration of effect sizes that have been estimated in various research studies (e.g., clinical studies for a particular agent in different clinical settings). However, to our knowledge, potential studies eligible for meta-analysis on this topic do not exist, and therefore, meta-analysis is not feasible. Secondly, some other factors, such as type of cancer (solid cancers vs. hematological malignancies), the number of approved indications reflecting the prevalence of the use of a particular TAA in the cancer population, type of mAb (e.g., mouse vs. humanized mAb) and the year of the initial approval might give us some further granularity of the data. However, the inclusion of additional variables in our study would decrease the robustness of our statistical analysis. Thirdly, since the availability of a CDx was defined as its use for all approved indications of a particular TAA, an alternative definition of the availability of a CDx might lead to different conclusions. However, a sensitivity analysis in which the availability of a CDx was defined as the use of a CDx for at least one approved indication of a particular TAA led to the same results. Finally, these findings might not fully apply to the emerging new classes of anticancer drugs (e.g., antibody–drug conjugates, immune checkpoint inhibitors, bispecific antibodies) that were underrepresented in our dataset. Due to their very small numbers, these new anticancer agents were grouped and analyzed together with other mAbs in our analysis.

## 5. Conclusions

While TAAs offer substantial therapeutic benefits to patients with various cancers, they are associated with safety concerns that require continuous scrutiny. The oncological community, regulatory bodies, and payers should be aware of the continuously evolving benefit–risk ratio of approved TAAs in the post-market setting. In general, there has been great improvement in the assessment of the benefit–risk ratio in the last two decades, from a subjective and inconsistent to a more structured, transparent, and consistent approach with a number of quantitative methods to incorporate preference weight from various stakeholders. However, while this development is encouraging, there is still more work to be carried out [33]. In everyday clinical practice, oncologists should regularly consult updated drug labels on the safety of TAAs, including those that have been on the market for a long time already. More rigorous evaluation of TAAs within clinical trials and in the real-world setting might lead to more predictable safety profiles.

## Figures and Tables

**Table 1:** A generalized linear mixed model for Warnings & Precautions.

Variable	Coef.	Confidence Int.	*p*-Value
Intercept	1.35	[1.05, 1.64]	<0.001
Time	0.06	[0.04, 0.08]	<0.001
Time^2^	−0.002	[−0.003, −0.001]	0.001
Drug type SMs (Ref. mAbs)	0.35	[0.006, 0.69]	0.042
CDx availability Yes (ref. No)	−0.15	[−0.54, 0.24]	0.440

Legend: W&Ps—Warnings & Precautions, SM—small molecule, mAb—monoclonal antibody, CDx—companion diagnostics for biomarkers, ref.—reference, coef.—coefficient, int.—interval.

**Table 2:** A generalized linear mixed model for Boxed Warnings.

Variable	Coef.	Confidence Int.	*p*-Value
Intercept	0.07	[1.05, 1.19]	0.90
Time	0.07	[0.02, 0.13]	0.008
Time^2^	−0.005	[−0.007, −0.002]	0.001
Drug type SMs (Ref. mAbs)	−3.11	[−4.60, −1.61]	<0.001
Availability of CDx Yes (ref. No)	0.22	[−1.33, 1.78]	0.77

Legend: BWs—black box warnings, SM—small molecule, mAb—monoclonal antibody, CDx—companion diagnostics for biomarkers, ref.—reference, coef.—coefficient, int.—interval.

## Data Availability

The data underlying this article are available on the Drugs@FDA website at https://www.fda.gov/drugs/drug-approvals-and-databases/about-drugsfda (accessed on 1 September 2023).

## Supplementary Materials

The following supporting information can be downloaded at: https://www.mdpi.com/article/10.3390/cancers17040584/s1, Figure S1: Distribution of the number of W&Ps for each targeted agent over time (in years); Figure S2: Distribution of BWs for each targeted agent over time (in years); Table S1: Characteristics of eligible targeted anticancer agents; Table S2. Results of sensitivity analysis for Warnings and Precautions; Table S3. Results of sensitivity analysis for Boxed Warning.
