# Supplementary material for: New Serious Safety Warnings for Targeted Anticancer Agents After Their Initial FDA Approval
Source: Cancers (Basel). 2025 Feb 8;17(4):584. doi: 10.3390/cancers17040584 (PMC11853166; doi:10.3390/cancers17040584)
Supplement: Supplementary file 1 [file cancers-17-00584-s001.zip › cancers-3448311-supplementary.pdf]

Supplementary Table S1: Characteristics of eligible targeted anticancer agents

Drug	Drug type	Availability of CDx	Date of initial approval	Date of last available label	Total number of W&Ps	Number of late W&Ps	Description of late W&Ps	Total number of BWs
rituximab	mAb	No	26.11.1997	17.12.2021	10	8	Bowel obstruction and perforation; Higher risk of infections; Renal toxicity; Hepatitis B virus reactivation**; Immunization (avoid live vaccines); Severe mucocutaneus reactions**; Progressive multifocal leukoencephalopathy**; Tumor lysis syndrome;	4
trastuzumab	mAb	Yes	25.09.1998	19.11.2018	4	1	Exacerbation of Chemotherapy-Induced Neutropenia;	3
imatinib mesylate	SM	No*	10.05.2001	19.08.2022	13	7	Severe congestive heart failure and left ventricular dysfunction; Hypereosinophilic cardiac toxicity; Hypothyroidism; Growth retardation in children and pre-adolescents; Tumor Lysis Syndrome; Higher risk of motor vehicle accidents; Renal Toxicity;	0
ibritumomab tiuxetan	mAb	No	19.02.2002	11.12.2018	5	2	Extravasation; Severe cutaneous and mucocutaneous reactions**	3
gefitinib	SM	Yes	05.05.2003	05.05.2021	6	4	Gastrointestinal perforation; Diarrhea; Ocular Disorders including Keratitis; Bullous and Exfoliative Skin Disorders;	0
bortezomib	SM	No	13.05.2003	04.11.2021	9	1	Thrombotic Microangiopathy;	0
cetuximab	mAb	No*	12.02.2004	24.09.2021	5	1	Hypomagnesemia and Accompanying Electrolyte Abnormalities;	2
bevacizumab	mAb	No	26.02.2004	18.09.2022	12	2	Venous Thromboembolic Events; Ovarian Failure;	0
erlotinib hydrochloride	SM	No*	18.11.2004	18.10.2016	9	0	/	0

Drug	Drug type	Availability of CDx	Date of initial approval	Date of last available label	Total number of W&Ps	Number of late W&Ps	Description of late W&Ps	Total number of BWs
sorafenib tosylate	SM	No	01.12.2005	07.09.2020	9	3	QT Prolongation; Drug-Induced Liver Injury; Impairment of Thyroid Stimulating Hormone Suppression in Differentiated Thyroid Cancer	0
dasatinib	SM	No	28.06.2006	08.02.2023	10	5	Pulmonary Arterial Hypertension; Severe Dermatologic Reactions; Tumor Lysis Syndrome; Effects on Growth and Development in Pediatric Patients; Hepatotoxicity;	0
panitumumab	mAb	Yes	27.09.2006	25.08.2021	5	1	Ocular Toxicities;	1
sunitinib malate	SM	No	26.01.2006	30.08.2020	14	8	Impaired Wound Healing; Osteonecrosis of the Jaw; Tumor Lysis Syndrome; Hypoglycemia; Proteinuria; Dermatologic Toxicities; Thrombotic microangiopathy; Reversible Posterior Leukoencephalopathy Syndrome;	1
lapatinib ditosylate	SM	Yes	13.03.2007	27.03.2022	7	1	Severe cutaneous reactions;	1
temsirolimus	SM	No	30.05.2007	23.03.2018	9	1	Proteinuria and nephrotic syndrome;	0
nilotinib hydrochloride	SM	Yes	29.10.2007	23.09.2019	11	4	Cardiac and Arterial Vascular Occlusive Events; Hemorrhage; Fluid Retention; Effects on Growth and Development in Pediatric Patients;	2
everolimus	SM	No	30.03.2009	01.02.2022	8	2	Severe Hypersensitivity Reactions; Angioedema;	0
pazopanib hydrochloride	SM	No	19.10.2009	13.12.2021	15	2	Interstitial Lung Disease/Pneumonitis; Tumor Lysis Syndrome;	1
ofatumumab	mAb	No	26.10.2009	30.08.2016	5	0	/	2
ipilimumab	mAb	No*	25.03.2011	15.02.2023	3	2	Infusion-Related Reactions; Complications of allogeneic hematopoietic stem cell transplantation;	0

Drug	Drug type	Availability of CDx	Date of initial approval	Date of last available label	Total number of W&Ps	Number of late W&Ps	Description of late W&Ps	Total number of BWs
vandetanib	SM	No	06.04.2011	28.03.2022	11	1	Impaired wound healing;	1
vemurafenib	SM	Yes	17.08.2011	18.05.2020	12	1	Dupuytren’s Contracture and plantar fascial fibromatosis;	0
brentuximab vedotin	mAb	No	19.08.2011	14.06.2023	11	1	Hyperglycemia;	1
crizotinib	SM	Yes	26.08.2011	14.07.2022	6	1	Gastrointestinal Toxicity;	0
ruxolitinib phosphate	SM	No	16.11.2011	31.01.2023	7	3	Major Adverse Cardiovascular Events; Thrombosis; Secondary Malignancies;	0
axitinib	SM	No	27.01.2012	22.09.2022	11	1	Major adverse cardiovascular events;	0
vismodegib	SM	No	30.01.2012	27.03.2023	2	2	Severe Cutaneous Adverse Reactions; Musculoskeletal Adverse Reactions;	0
pertuzumab	mAb	Yes	08.06.2012	16.01.2020	3	0	/	1
carfilzomib	SM	No	20.07.2012	30.06.2022	14	1	Progressive Multifocal Leukoencephalopathy;	0
ziv - aflibercept	mAb	No	03.08.2012	05.06.2020	10	0	/	0
bosutinib monohidrate	SM	No	04.09.2012	20.04.2023	6	1	Cardiovascular Toxicity;	0
regorafenib	SM	No	27.09.2012	10.12.2020	9	0	/	1
cabozantinib s-malate	SM	No	29.11.2012	13.02.2023	14	5	Hepatotoxicity; Adrenal Insufficiency; Thyroid Dysfunction; Hypocalcemia; Diarrhea;	0
ponatinib hydrochloride	SM	No	14.12.2012	15.02.2022	15	0	/	4
ado-trastuzumab emtansine	mAb	Yes	22.02.2013	02.02.2022	7	0	/	2

Drug	Drug type	Availability of CDx	Date of initial approval	Date of last available label	Total number of W&Ps	Number of late W&Ps	Description of late W&Ps	Total number of BWs
dabrafenib	SM	Yes	29.05.2013	26.05.2023	9	1	Hemophagocytic Lymphohistiocytosis;	0
trametinib	SM	Yes	29.05.2013	26.05.2023	11	1	Hemophagocytic Lymphohistiocytosis;	0

Legend: W&Ps – Warnings and precautions, BWs – Black Box Warnings, mAb – monoclonal antibody, SM – small molecule, CDx – companion diagnostics for biomarkers

*Targeted anticancer agents that do not have available companion diagnostics for all indications

** Late Black Box Warnings

Supplementary Table S2. Results of sensitivity analysis for Warnings and Precautions

Variable	Coef.	Confidence int.	P-value
**Intercept**	1.42	[1.13, 1.70]	<0.001
**Time**	0.06	[0.04, 0.08]	< 0.001
**$Time^2$**	-0.002	[-0.003, -0.001]	0.001
**Drug type**			
SMs (Ref. mAbs)	0.32	[0.01, 0.63]	0.04
**CDx availability**			
Yes (Ref. No)	-0.24	[-0.57, 0.08]	0.14

Legend: W&Ps – Warnings and precautions, SM – small molecule, mAb – monoclonal antibody, CDx – companion diagnostics for biomarkers, ref. – reference, coef. – coefficient, int. – interval

Supplementary Table S3. Results of sensitivity analysis for Boxed

**Warning Variable**	**Coef.**	**Confidence int.**	**P-value**
**Intercept**	0.37	[-0.75, 1.48]	0.51
**Time**	0.07	[0.02, 0.13]	0.008
**Time**^{2}	-0.005	[-0.007, -0.002]	0.001
**Drug type**			
SMs (Ref. mAbs)	-3.21	[-4.60, -1.84]	<0.001
**Availability of CDx**			
Yes (Ref. No)	-0.45	[-1.88, 0.97]	0.53

Legend: BWs – black box warnings, SM – small molecule, mAb – monoclonal antibody, CDx – companion diagnostics for biomarkers, ref. – reference, coef. – coefficient, int. - interval

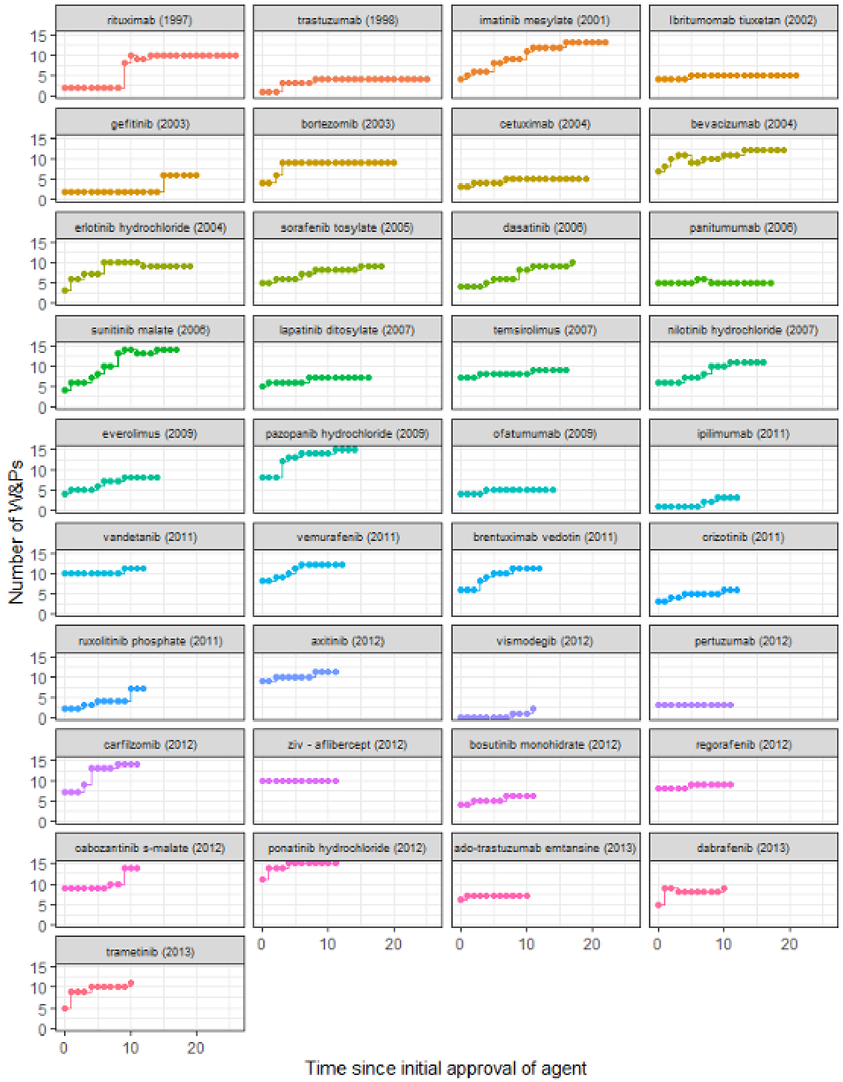

Supplementary Figure S1: Distribution of the number of W&Ps for each targeted agent over time (in years)

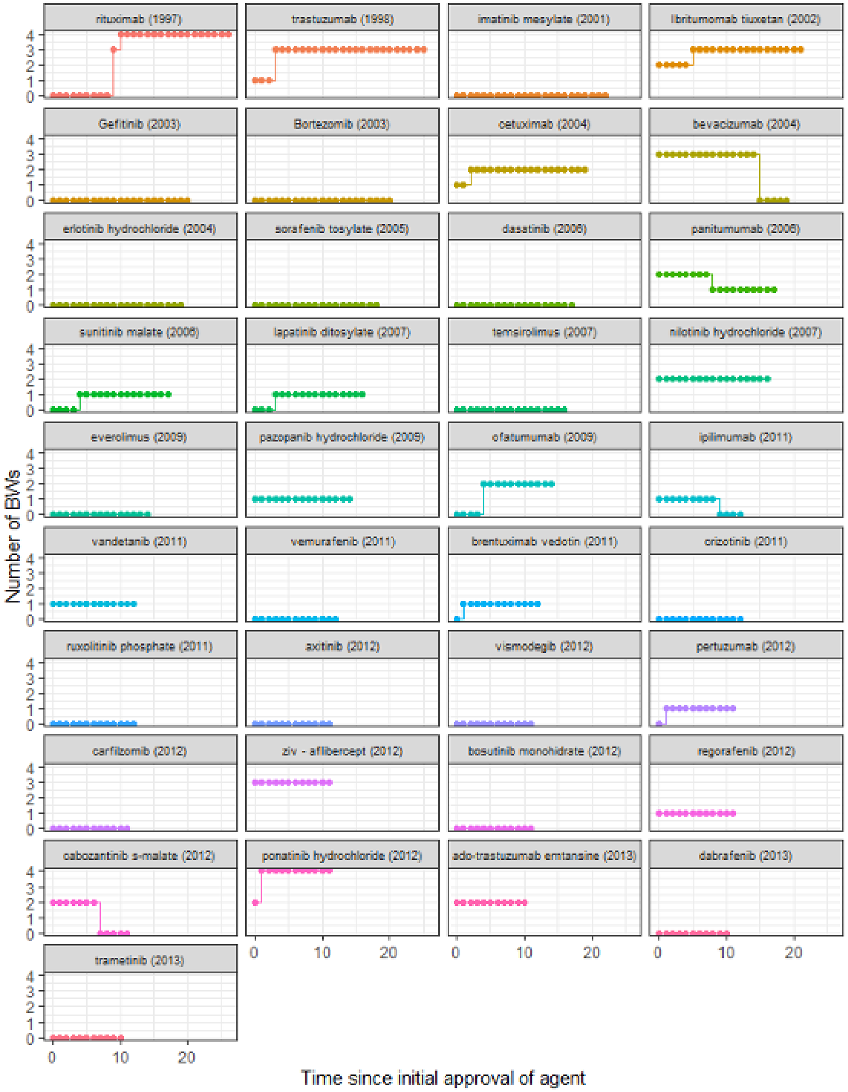

Supplementary Figure S2: Distribution of BWs for each targeted agent over time (in years)
